# Overexpression of NT-3 in the hippocampus suppresses the early phase of the adult neurogenic process

**DOI:** 10.3389/fnins.2023.1178555

**Published:** 2023-07-27

**Authors:** Nanami Kasakura, Yuka Murata, Asuka Shindo, Shiho Kitaoka, Tomoyuki Furuyashiki, Kanzo Suzuki, Eri Segi-Nishida

**Affiliations:** ^1^Department of Biological Science and Technology, Faculty of Advanced Engineering, Tokyo University of Science, Tokyo, Japan; ^2^Department of Pharmacology, School of Medicine, Hyogo Medical University, Hyogo, Japan; ^3^Division of Pharmacology, Kobe University Graduate School of Medicine, Kobe, Japan

**Keywords:** NT-3, hippocampus, neurogenesis, dentate gyrus, maturation, AAV

## Abstract

The dentate gyrus (DG) of the hippocampus regulates stress-related emotional behaviors and ensures neurogenesis throughout life. Neurotrophin-3 (NT-3) is a neurotrophic factor that regulates neuronal differentiation, survival, and synaptic formation in both the peripheral and central nervous systems. NT-3 is expressed in the adult DG of the hippocampus; several chronic stress conditions enhance NT-3 expression in rodents. However, functional modulation of the adult DG by NT-3 signaling remains unclear. To directly investigate the impact of NT-3 on DG function, NT-3 was overexpressed in the hippocampal ventral DG by an adeno-associated virus carrying NT-3 (AAV-NT-3). Four weeks following the AAV-NT-3 injection, high NT-3 expression was observed in the ventral DG. We examined the influence of NT-3 overexpression on the neuronal responses and neurogenic processes in the ventral DG. NT-3 overexpression significantly increased the expression of the mature DG neuronal marker calbindin and immediate early genes, such as *Fos* and *Fosb*, thereby suggesting DG neuronal activation. During neurogenesis, the number of proliferating cells and immature neurons in the subgranular zone of the DG significantly decreased in the AAV-NT-3 group. Among the neurogenesis-related factors, *Vegfd, Lgr6, Bmp7,* and *Drd1* expression significantly decreased. These results demonstrated that high NT-3 levels in the hippocampus regulate the activation of mature DG neurons and suppress the early phase of neurogenic processes, suggesting a possible role of NT-3 in the regulation of adult hippocampal function under stress conditions.

## Introduction

The hippocampus is a limbic structure implicated in the regulation of stress responses and pathophysiology of depression. The dentate gyrus (DG) is a part of the hippocampus that receives cortical inputs from the entorhinal cortex and projects them to the CA3 pyramidal cells. The DG is one of the few areas of the mammalian brain in which adult neurogenesis occurs (Ming and Song, 2005; Kempermann et al., 2015). The neurogenic processes include proliferation, differentiation, survival, and functional maturation. These processes are positively or negatively regulated by external stimuli, such as learning, exercise, antidepressant treatment, and stress (Warner-Schmidt and Duman, 2006; Zhao et al., 2008; Leschik et al., 2021; Segi-Nishida and Suzuki, 2022).

Neurotrophin-3 (NT-3) is a neurotrophic factor that is known to regulate neuronal survival, differentiation, and synaptic function in both the peripheral and central nervous systems (Ernfors et al., 1994; Barbacid, 1995). In hippocampal neuronal cultures, NT-3 induced the expression of calbindin, a mature neuronal marker in the DG, and c-fos, a product of an immediate early gene (IEG) (Collazo et al., 1992), suggesting the effect of NT-3 on neuronal maturation and activation. In developmental neural stem cell culture, certain reports have demonstrated that NT-3 inhibits mitosis and lengthens the cell cycle (Ghosh and Greenberg, 1995; Lukaszewicz et al., 2002; Simpson et al., 2003), while others have reported that NT-3 promotes cell division and survival rate (Barnabe-Heider and Miller, 2003; Lu et al., 2011).

Although several studies have been reported, the role of NT-3 in adult neurogenesis *in vivo* remains unclear. One report revealed that the differentiation and survival, but not proliferation, of neuronal precursor cells were impaired in the DG of mice lacking NT-3 (Shimazu et al., 2006). However, another report demonstrated that the proliferation of neural stem cells in the subventricular zone, another neurogenic region in adults, was increased in NT-3 heterozygous mice, suggesting a suppressive role of NT-3 in neural stem cell proliferation (Delgado et al., 2014).

In rats, NT-3 is highly expressed in the central nervous system during development; however, its expression declines as these regions mature (Maisonpierre et al., 1990). Nevertheless, high expression of NT-3 is maintained in the hippocampus even in adult rats (Katoh-Semba et al., 1996). Although NT-3 is highly expressed in the DG compared to other hippocampal regions, such as CA1 and CA3 (Smith et al., 1995), its effects of NT-3 on the function of mature neurons in the DG remain unclear. We previously reported that chronic administration of antidepressants, such as fluoxetine, or electroconvulsive seizures, decreases NT-3 expression in the DG of adult mice (Imoto et al., 2017). Conversely, a previous study demonstrated that NT-3 expression was increased in the hippocampus following chronic unpredictable mild stress (Jiang et al., 2014). In addition, corticosterone administration increased NT-3 expression in the DG (Smith et al., 1995). However, whether increased NT-3 alters the neurogenic processes and neuronal maturation in the DG *in vivo* remains unclear.

The hippocampus reportedly plays functionally distinct roles in the dorsal and ventral regions (Fanselow and Dong, 2010), and both the regions respond to stress (Hawley et al., 2012; Maras et al., 2014). While the dorsal region is thought to be involved in stress-induced learning and memory changes, the ventral region contributes to stress response and emotions such as anxiety. Specifically, increased neurogenesis in the ventral DG reportedly contributes to stress resilience (Anacker et al., 2018), suggesting that identification of the key signaling pathway in the ventral DG would be important to understand the underlying mechanisms of stress response and emotion. While NT-3 is expressed in both the dorsal and ventral DG, we hypothesized that modulation of the NT-3 signals could alter the adult neurogenic processes and neuronal responses in the ventral DG.

In this study, we investigated the influence of high levels of NT-3 expression on the adult neurogenic processes and neuronal responses in the ventral DG using an adeno-associated virus (AAV) expression system. Elucidation of the function of NT-3 in the DG *in vivo* would result in a better understanding of the role of NT-3 in adult neurogenesis, as well as in the stress response.

## Materials and methods

### Experimental animals

Seven-to eight-week-old male C57BL/6 N mice (23–25 g) were purchased from Japan SLC (RRID5295404; Hamamatsu, Japan). The mice were housed in groups of six per cage. All the mice were housed under standard conditions (24 ± 2°C, 55% ± 5% humidity) with a 12 h light/dark cycle and *ad libitum* access to water and food. Weight gain of each mouse was recorded during the experiments to monitor the physical conditions of the animals. All the mice were habituated for longer than 1 week before the experimental procedures were performed. Animal use and procedures were performed according to the guidelines prescribed by the National Institute of Health and approved by the Animal Care and Use Committee of the Tokyo University of Science (approval number K20009, K21007, K22007).

### AAV preparation and hippocampal administration

For the generation of AAV carrying NT-3 (AAV-NT-3) vector plasmids, an NT-3 coding sequence was amplified using polymerase chain reaction (PCR) from mouse hippocampal cDNA and inserted into the pcDNA3 plasmid (Thermo Fisher Scientific, Waltham, MA, USA). A CMV-NT-3 cassette was inserted into the MluI-EcoRV site of the pAAV-EF1α-DIO EYFP vector (Addgene #27056). For the generation of AAV carrying enhanced green fluorescent protein (AAV-EGFP), the NT-3 gene in the AAV-NT-3 was replaced with a gene encoding EGFP. To generate AAV-EF1α-Emerald-GFP (EmGFP), an EmGFP sequence was amplified using PCR from the pcDNA 6.2-GW/EmGFP-miR (Thermo Fisher Scientific) and inserted into the KpnI-EcoRV site of the pAAV-EF1α-DIO EYFP vector (Addgene #27056). AAV2/rh10 particles were produced as previously described with minor modifications (Shinohara et al., 2018). Briefly, the AAV-rep2/caprh10 expression plasmid (Addgene #112866), pHelper vector (#240071, Agilent Technologies, Santa Clara, CA), and the constructed AAV-vector plasmid were transfected into the AAV-293 cells (Agilent, #240073) using Lipofectamine 2000 (Thermo Fisher Scientific). The transfected AAV-293 cells were collected, resuspended in artificial cerebrospinal fluid, frozen, and thawed four times 3 days after transfection. Cell debris was eliminated by centrifugation at 10,000× *g* for 10 min, and the supernatant containing the virus was collected. The viral suspension was incubated with benzonase (Merck, Darmstadt, Germany) to degrade the residual DNA. The prepared AAV solution was aliquoted and stored at −80°C. The AAV titers were quantified using PCR. Under anesthesia with medetomidine (0.3 mg/kg, ZENOAQ, Fukushima, Japan), midazolam (4 mg/kg, Sandoz, Tokyo, Japan), and butorphanol (5 mg/kg, Meiji Seika Pharma, Tokyo, Japan) mixture, the mouse brain was fixed with stereotaxic instruments (Narishige, Tokyo, Japan). An AAV solution of 500 nL per injection site (2.5 × 10^8^ copies) was stereotaxically injected at two sites in each hemisphere into the DG using a PV-820 Pneumatic PicoPump (World Precision Instruments, Hessen, Germany) through a glass micropipette (World Precision Instruments) made with a PN-30 micropipette puller (Narishige). The stereotaxic coordinates were targeted to the ventral DG:3.2 mm posterior to the bregma, 2.7 mm lateral to the midline, and 3.0 mm ventral to the skull surface at the bregma according to a mouse brain atlas (Paxinos and Franklin, 2012). To visually detect viral infection, 5 × 10^7^ copies of AAV-EF-1α-EmGFP were co-injected with AAV-NT-3 and AAV-EGFP in the DG of the hippocampus. The data were excluded from the analysis if fluorescence was not observed in the DG.

### RNA extraction and real-time PCR

The mice were decapitated, and coronal brain slices (1 mm) were cut using a tissue slicer. To isolate the ventral DG, the hippocampal DG from the coronal brain slice of bregma −2.0 mm to −4.0 mm (Paxinos and Franklin, 2012) was dissected under a stereoscopic microscope. Total RNA was extracted using the Reliaprep RNA Cell Miniprep System (Promega, Madison, WI, USA) and reverse transcribed using ReverTra Ace (Toyobo, Osaka, Japan) followed by real-time PCR using the StepOne system (Applied Biosystems, Foster City, CA) and Thunderbird SYBR qPCR mix (Toyobo). The expression levels of each gene were quantified using standardized external dilutions. The relative expression levels of the target genes were normalized to those of 18S rRNA. The specificity of each primer set was confirmed by melt-curve analysis and the product size was examined using gel electrophoresis. Primer sequences for each gene are listed in Table 1.

**Table 1:** List of primers used for qPCR analysis.

Gene	Forward (5′ to 3′)	Reverse (5′ to 3′)
*Ntf3*	AGTTTGCCGGAAGACTCTCTC	GGGTGCTCTGGTAATTTTCCTTA
*Trkc*	CTGAGTGCTACAATCTAAGCCC	CACACCCCATAGAACTTGACAAT
*Calb1*	TCTGGCTTCATTTCGACGCTG	ACAAAGGATTTCATTTCCGGTGA
*Fos*	CAGAGCGGGAATGGTGAAGA	TCGGTGGGCTGCCAAAATAA
*Fosb*	TTTTCCCGGAGACTACGACTC	GTGATTGCGGTGACCGTTG
*Arc*	AGCGGGACCTGTACCAGAC	AGCTGCTCCAGGGTCTTG
*Egr1*	TATGAGCACCTGACCACAGAG	GCTGGGATAACTCGTCTCCA
*Bdnf*	GACAAGGCAACTTGGCCT AC	ACTGTCACACACGCTCAGCTC
*Npas4*	CAGATCAACGCCGAGATTCG	CACCCTTGCGAGTGTAGATGC
*Vegfa*	GCTGCACCCACGACAGAAG	CGCTGGTAGACATCCATGAAC
*Figf/Vegfd*	TTGAGCGATCATCCCGGTC	GCGTGAGTCCATACTGGCAAG
*Wnt2*	CTCGGTGGTGGAATCTGGCTCTG	CACATTGTCACACATCACCCT
*Fzd6*	TCTGCCCCTCGTAAGAGGAC	GGGAAGAACGTCATGTTGTAAGT
*Lgr6*	GAGGACGGCATCATGCTGTC	GCTCCGTGAGGTTGTTCATACT
*Bmp2*	GGGACCCGCTGTCTTCTAGT	TCAACTCAAATTCGCTGAGGAC
*Bmp4*	TTCCTGGTAACCGAATGCTGA	CCTGAATCTCGGCGACTTTTT
*Bmp7*	ACGGACAGGGCTTCTCCTAC	ATGGTGGTATCGAGGGTGGAA
*Drd1*	ACAGCAGCCCCTCCGATAG	GTTAGACCTGGGCAGATGAAG
*Iba1/Aif1*	ATCAACAAGCAATTCCTCGATGA	CAGCATTCGCTTCAAGGACATA

### Immunoblot

The mice were decapitated; coronal brain slices (1 mm) were cut using a tissue slicer and the ventral DG of the hippocampus was dissected under a stereoscopic microscope. The isolated DG was homogenized in a protein lysis buffer (50 mM Tris HCl pH 7.4, 150 mM NaCl, 0.5% sodium deoxycholate, 2 mM EDTA, 1 mM EGTA, 1% TritonX-100) containing protease inhibitor cocktail (Nacalai tesque, Kyoto, Japan) on ice and centrifuged at 16,000× *g* for 20 min at 4°C. Supernatants containing 10 μg proteins were separated on 12.5% sodium dodecyl sulfate (SDS)-polyacrylamide gel by electrophoresis and transferred onto the polyvinyl difluoride membrane. The membrane was first blocked with 5% skim milk in Tris-buffered saline for 1 h at room temperature and subsequently incubated with rabbit anti-NT-3 antibody (1:4500, kindly provided by Drs. Furuichi and Semba; Katoh-Semba et al., 1996), rabbit anti-GFP monoclonal antibody (1:500, #598, Medical and biological laboratories, Japan, RRID:AB_591819), or mouse anti-β-actin monoclonal antibody (1:4000, MA5-15739, Thermo Fisher Scientific, RRID:AB_10979409) at 4°C overnight. After washing, the membrane was incubated with horseradish peroxidase conjugated goat anti-rabbit IgG (1:5000, Jackson ImmunoResearch, Jackson ImmunoResearch, West Grove, PA, 111–035-144, RRID:AB_2307391) or horse anti-mouse IgG (1:2000, Vector Laboratories Inc., Burlingame, CA, PI-2000, RRID:AB_2336177) secondary antibody for 1 h at room temperature, and the bands were visualized with EzWestLumiOne (WSE-7110, ATTO, Tokyo, Japan) by LAS-4000 (GE Healthcare Life Science, Pittsburgh, PA).

### Sample isolation, immunohistochemistry and 5′-ethynyl-2′-deoxyuridine (EdU) detection

#### EdU and 5′-bromo-2′-deoxyuridine (BrdU) administration

To label dividing cells during the S-phase of mitosis, mice were administered BrdU (150 mg/kg, intraperitoneal [i.p.], Nacalai Tesque) twice a day, 2 to 4 days before the AAV injection, or EdU (100 mg/kg, i.p., Abcam, Cambridge, UK) 2 h before sacrifice.

#### Brain isolation and section preparation

For NT-3 immunostaining, mice were deeply anesthetized and transcardially perfused with cold saline followed by 0.25% glutaraldehyde and 2% paraformaldehyde in 0.1 M phosphate buffer (pH 7.4) and were post-fixed at 4°C for 24 h. For other immunostaining procedures, mice were perfused with cold saline followed by 4% paraformaldehyde in 0.1 M phosphate buffer (pH 7.4), and were post-fixed at 4°C for 48 h. The brains were cryoprotected in 20% sucrose for 48 h, then 30% sucrose for 24 h and stored at −80°C until further use. Serial sections (30 μm thick) were cut through the entire hippocampus using a cryostat (Leica 1510, Leica Microsystems, Wetzlar, German) and stored in 30% glycerol and 30% ethylene glycol in 0.02 M phosphate buffer (pH 7.4) at −20°C until staining.

#### Immunohistochemistry and EdU detection

The sections were washed with phosphate-buffered saline (PBS) and blocked using 10% equine serum (Cytiva, Tokyo, Japan, SH30074) in PBS containing 0.3% Triton X-100 (PBST) at room temperature for 60 min, followed by overnight incubation with rabbit anti-NT-3 (1:450), rabbit anti-doublecortin (DCX) (1:1000; Cell Signaling Technology, Danvers, MA, #4604, RRID:AB _561007), rabbit anti-calbindin (1:8000; Swant, Marly, Switzerland, CB38, AB_10000340), rabbit anti-FosB (1:1000; Abcam, ab184938, RRID: AB_2721123), mouse anti-NeuN (1:1000, Merck Millipore, Burlington, MA, MAB377, RRID: AB_2298772), rabbit anti-TrkC (1:1600, Cell Signaling Technology, #3376, RRID: AB_2155283), rabbit anti-pErk1/2 (1:200, Cell Signaling Technology, #4370, RRID:AB_2315112), rabbit anti-pPLC γ1 (1:100,Cell Signaling Technology #8713, RRID:AB_10890863), and rabbit anti-Iba1 (1:4000; Wako, Okasa, Japan, 019–19,741, RRID: AB_839504) at 4°C. For BrdU immunostaining, the sections were incubated in 50% formamide in 2 × saline sodium citrate buffer for 2 h at 60°C, followed by incubation in 2 M hydrogen chloride at 37°C for 30 min and neutralized with 0.1 M boric acid (pH 8.5) at room temperature for 10 min before blocking. After blocking, the sections were incubated overnight with monoclonal rat anti-BrdU (1:8000; Abcam, ab6326, RRID:AB_305426) at 4°C. After washing with PBST, the sections were incubated using donkey anti-rabbit IgG antibody conjugated with Alexa Fluor 555 (1:300 or 1:1000, Thermo Fisher Scientific, A-31572, RRID:AB_162543), rabbit anti-mouse IgG antibody conjugated with Alexa Fluor 555 (1:300, Thermo Fisher Scientific, A-21427, RRID:AB_2535848) and donkey anti-rat IgG antibody conjugated with Alexa Fluor 555 (1:1000, Abcam, ab150154, RRID:AB_2813834) for 60 min. After washing with PBST, the sections were incubated with 4′,6-diamidino-2-phenylindole (DAPI; 1:10000; Merck Millipore) and mounted on slides with Mowiol (Merck Millipore) after washing with PBS. For Iba1 and pERK staining, after washing with PBST, sections were incubated with biotinylated goat anti-rabbit IgG (1:200; Vector Laboratories Inc., Burlingame, CA, BA1000, RRID: AB_2313606) for 60 min. The sections were washed with PBST and incubated with the ABC Vectastain Kit (Vector), and antigen detection was performed with 0.02% 3,3′-diaminobenzidine (Wako, 049–22,831) staining. After washing, the sections were mounted on slides with Entellan New (Merck Millipore). EdU Detection was performed using Click-iT EdU imaging kit (Thermo Fisher Scientific), followed by mitotic staining with Alexa Fluor 555. *In situ* Apoptosis Detection kit was used for evaluating the levels of apoptosis according to the manufacturer’s instructions (TaKaRa, MK500, Shiga, Japan).

### Quantification of EdU-, BrdU-, doublecortin-, calbindin-, Iba1-, TrkC-, and FosB-positive cells

For EdU-labeled cell quantification in the dorsal and ventral DG, we used modified unbiased stereology protocol, which has been able to successfully quantify thymidine-analog labeling (West et al., 1991; Malberg et al., 2000). Briefly, every sixth section of the entire DG (30 μm) was selected to ensure that same cells will not be counted in two sections with the first half defined as the dorsal DG and the second half as the ventral DG. EdU-positive (+) cells were counted in the subgranular zone (SGZ) in either dorsal or ventral DG for cell proliferation using a fluorescent microscope (BZX-700, Keyence, Osaka, Japan). The SGZ was defined as a two cell-body width zone along the border of the granule cell layer and hilus. The sum of cell counts was multiplied by 6 to provide an estimate of the total number of EdU (+) cells in each DG region.

For BrdU-labeled cell quantification, 3–6 sections of the ventral DG were photographed using a microscope. The number of BrdU (+) cells is presented as the number of cells per 10,000 μm^2^ area in the granule cell layer (GCL) in the DG using computer-assisted image analysis (ImageJ, NIH, Bethesda, MD, USA). For neuronal differentiation of BrdU-labeled cells, six slices were analyzed to determine if BrdU-labeled cells were colabeled with NeuN. For DCX (+) cell quantification, two or three sections of the ventral DG were photographed using a microscope. DCX (+) cells, in which dendrites did not reach the molecular layer, were subcategorized as DCX (+) cells with short dendrites, whereas DCX (+) cells, in which dendrites reached the molecular layer and had complex processes, were subcategorized as DCX (+) cells with long and branched dendrites. The number of DCX (+) cells was measured as the number of cells per 100 μm along the SGZ in the DG using ImageJ software. To quantify calbindin and TrkC immunoreactivity, three sections of the DG were photographed under a microscope. The molecular layer or GCL of the DG was set as the region of interest (ROI) and each ROI was measured. The average signal intensity of immunoreactivity in the molecular layer or GCL in the DG was quantified using ImageJ software. To measure the Iba1 immune-positive area, the grayscale image was converted into a binary image, and the Iba1 immune-positive area was measured within the ROI. To quantify FosB immunoreactivity, three sections of the ventral DG were photographed under a microscope. The number of FosB (+) cells is presented as the number of cells per 10,000 μm^2^ area in the GCL in the DG using computer-assisted image analysis (QuPath, Bankhead et al., 2017).

### Statistical analyses

All the data are presented as the mean ± standard error of mean. Statistical analysis was performed using the unpaired Student’s *t*-test or Welch’s *t*-test. Statistical significance was set at *p* < 0.05. Before performing *t*-test, normality of the data was examined using the Shapiro–Wilk test. If the data did not pass the normality test, a nonparametric test, the Mann–Whitney U test, was performed. Detailed statistical data are presented in Supplementary Table S1. All the analyses were performed using PRISM 9 software (GraphPad, San Diego, CA).

## Results

### Overexpression of NT-3 in the DG of the hippocampus

**Figure 1:**
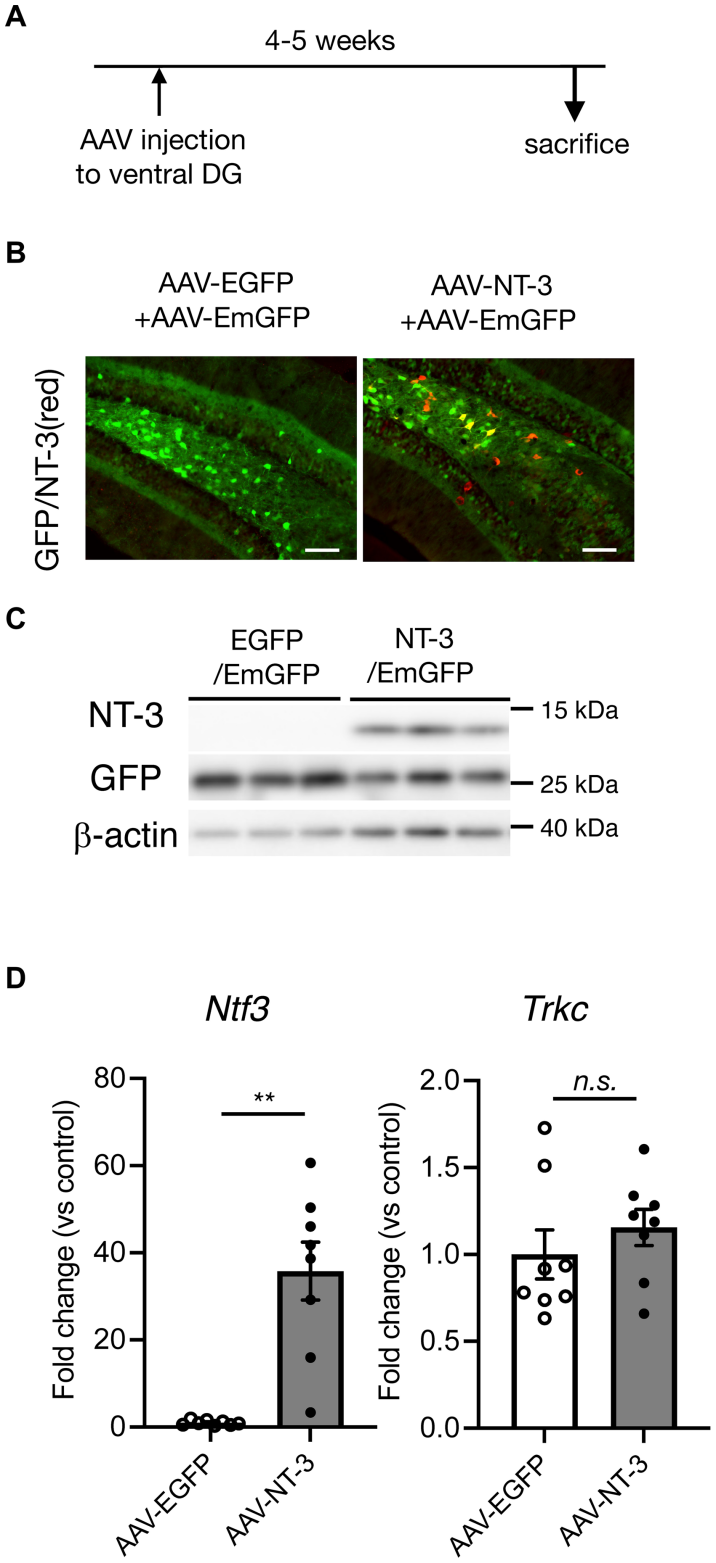
Overexpression of neurotrophin-3 (NT-3) in the DG of the hippocampus by AAV carrying NT-3 injection. **(A)** Time course of the experimental procedure. **(B)** The expression of GFP (green) and NT-3 (red) in the DG 4–5 weeks following AAV injection. Scale bars: 100 μm. **(C)** Confirmation of NT-3 protein overexpression in the DG. Typical immunoblots for anti-NT-3, anti-GFP, and anti-β-actin were shown. Each lane indicates different DG samples 4–5 weeks following AAV injection. **(D)** The confirmation of NT-3 mRNA overexpression (left, *p* = 0.0012) and the effect of NT-3 overexpression on the gene expression of *Trkc* (right, *p* = 0.3282) in the ventral DG. *n* = 8 each for the AAV-enhanced GFP group and AAV-NT-3 group. Data are expressed using dot plots and means ± standard error of mean. **p < 0.01. n.s., not significant.

To examine the influence of NT-3 on adult neurogenesis in the DG, we used AAVrh10-CMV-NT-3 virus (AAV-NT-3) for NT-3 overexpression or AAVrh10-CMV-EGFP virus (AAV-EGFP) for control. To visually confirm viral infection, 10% AAVrh10-EF1α-EmGFP was mixed with these vectors and injected into the ventral DG of the hippocampus. Four to 5 weeks after viral injection, GFP expression was observed in the GCL and hilus region of the DG in both the groups (Figures 1A,B). In the GCL, most of the GFP-positive cells were neuronal marker NeuN-positive and immature neuronal marker DCX-negative (Supplementary Figure 1A), suggesting that the cells overexpressing NT-3 in the GCL were mostly mature neurons. However, strong immunostaining signals for the anti-NT-3 antibody were detected, especially in the hilus of the DG in the AAV-NT-3 group (Figure 1B). A robust increase in the NT-3 expression was confirmed by immunoblotting and real-time PCR analysis (Figures 1C,D, left), while no effect was observed in the NT-3 receptor *Trkc* expression by NT-3 overexpression (Figure 1D, right).

Since a high amount of NT-3 could exert a toxic effect, we evaluated the levels of microglial activation and apoptotic cell death in NT-3 overexpression. No difference was observed in the immune-positive area of Iba1 immunostaining in the molecular layer between the AAV-EGFP and AAV-NT-3 groups (Supplementary Figures 1B,C). We further quantified *Iba1(Aif1)* mRNA expression in the DG; however, these were similar in both groups (Supplementary Figure 1D). In addition, few apoptosis-positive signals were detected in the GCL and hilus regions in both groups (Supplementary Figure 1E).

### The responses of DG neurons to high NT-3 expression

**Figure 2:**
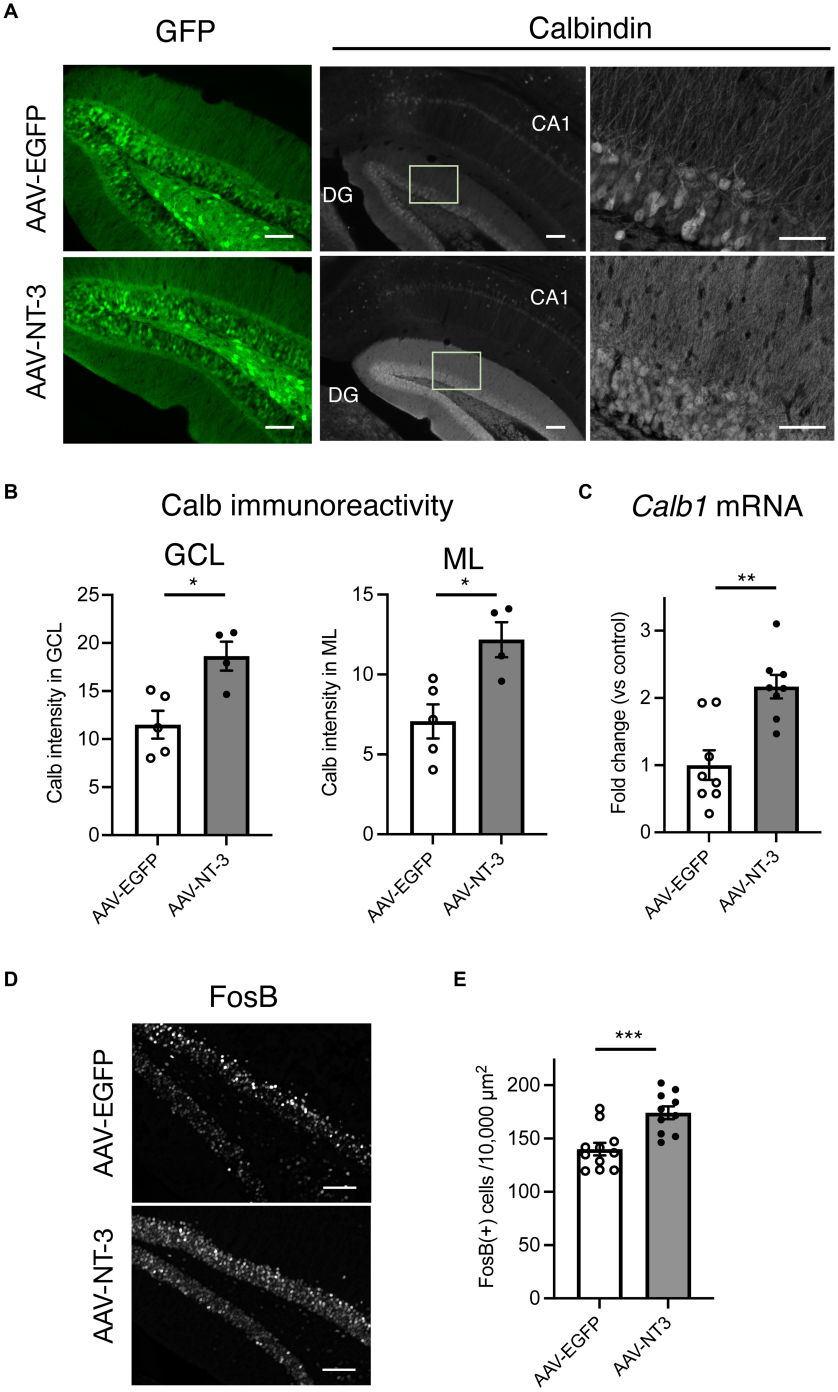
The response of hippocampal ventral DG neurons to high NT-3 expression. **(A)** The effect of NT-3 overexpression on the expression of mature neuronal marker, calbindin, in the ventral DG. Representative coronal images for GFP (left, scale bars: 100 μm) and anti-calbindin immunostaining (middle, scale bars: 100 μm) in the ventral DG are shown. The images on the right show the squares in the middle images (scale bars: 50 μm). **(B)** Quantification of the intensity of calbindin immunoreactivity in the granule cell layer (GCL, *p* = 0.0117) and molecular layer (ML, *p* = 0.0131). *n* = 5 for AAV-EGFP group and *n* = 4 AAV-NT-3 group. **(C)** The effect of NT-3 overexpression on the gene expression of *Calb1* (*p* = 0.001) in the ventral DG. *n* = 8 each for AAV-EGFP group and AAV-NT-3 group. **(D)** The expression of FosB in the ventral DG. Scale bars: 100 μm. **(E)** Quantification of FosB (+) cell numbers in the GCL. *p* = 0.0007, *n* = 11 for AAV-EGFP group and *n* = 10 AAV-NT-3 group. Data are expressed using dot plots and means ± standard error of mean. *p < 0.05; **p < 0.01; ***p < 0.001.

**Figure 3:**
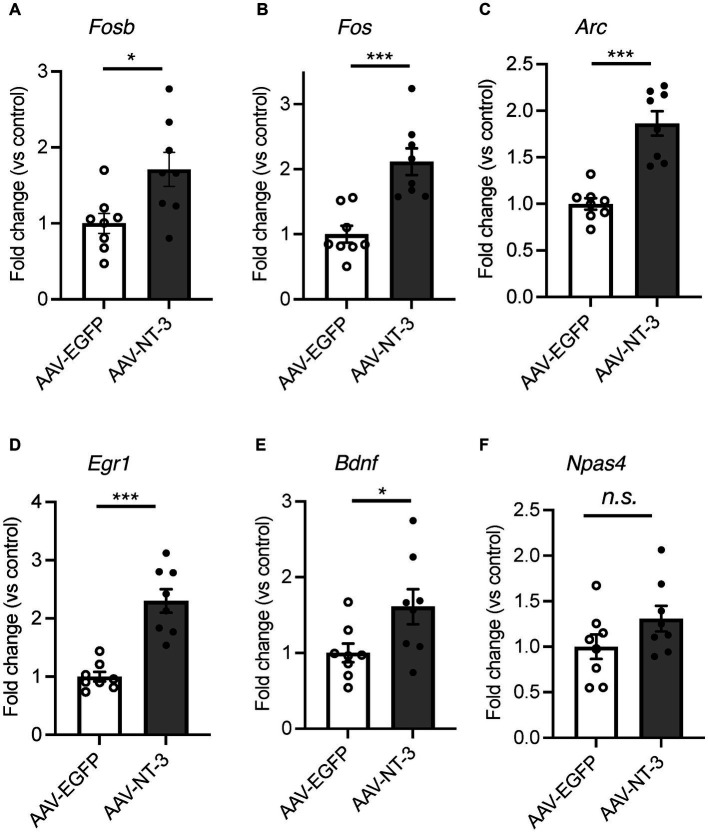
The effect of NT-3 overexpression on the gene expression of IEGs in the ventral DG. Expression of **(A)** *Fosb* (*p* = 0.0163), **(B)** *Fos* (*p* = 0.0004), **(C)** *Arc* (*p* < 0.0001), **(D)** *Egr1* (*p* = 0.0002), **(E)** *Bdnf* (*p* = 0.0358) and **(F)** *Napas4* (*p* = 0.1349) are shown. *n* = 8 each for AAV-EGFP group and AAV-NT-3 group. Data are expressed using dot plots and means ± standard error of mean. *p < 0.05; ***p < 0.001. n.s., not significant.

In cultured hippocampal neurons, NT-3 increases the expression of calbindin and c-fos (Collazo et al., 1992; Boukhaddaoui et al., 2001). We examined the influence of high NT-3 levels on calbindin expression in mature neurons of the ventral DG. The calbindin immunoreactivity signals in the GCL and molecular layer of the ventral DG were significantly enhanced in the AAV-NT-3 group (Figures 2A,B). We also confirmed that the mRNA expression of *Calb1* in the ventral DG significantly increased in the AAV-NT-3 group (Figure 2C). We subsequently examined whether neuronal activation in the DG was modulated by NT-3 overexpression. FosB, including ΔFosB, is an indicator of prolonged cellular activity (Nestler et al., 1999). Therefore, we performed immunohistochemical analysis using FosB to evaluate the neuronal activity in the ventral DG. FosB-positive cells in the GCL were significantly increased in the AAV-NT-3 group (Figures 2D,E). We also examined the gene expression in several types of IEGs in the ventral DG. NT-3 overexpression moderately, but significantly, increased the expression of *Fosb, Fos*, *Arc*, *Egr1*, and *Bdnf*, but not *Npas4* (Figure 3). These results suggest that NT-3 signaling directly or indirectly activates mature neurons in the GCL *in vivo*.

### The influence of high NT-3 expression on the early phase of the neurogenic process in the DG of the hippocampus

**Figure 4:**
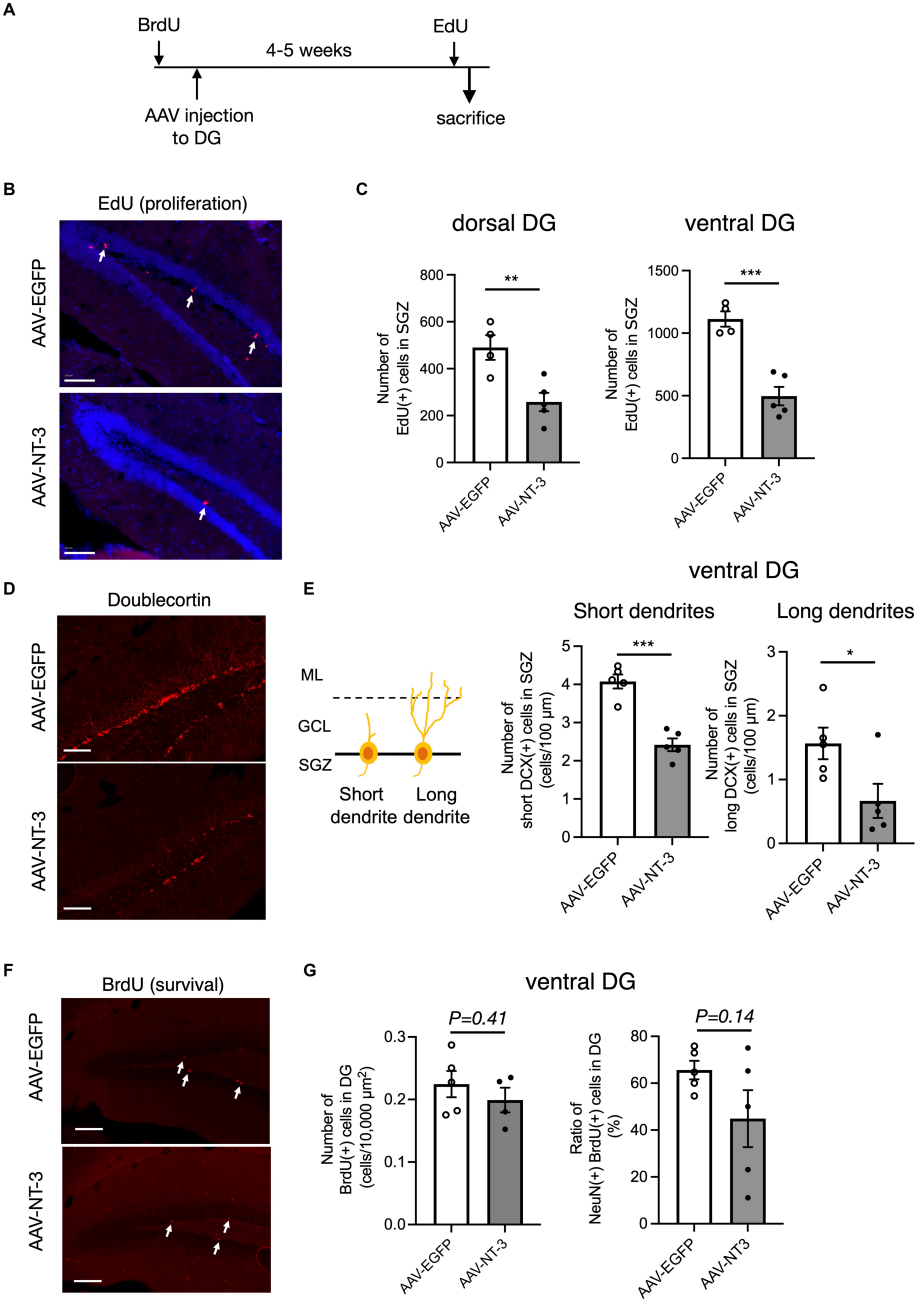
The effect of NT-3 overexpression on early phase of neurogenic process in the DG of the hippocampus. **(A)** Time course of the experimental procedure. **(B)** The effect of NT-3 overexpression on proliferation in the DG. Representative coronal images for EdU detection in the DG are shown. The arrows represent EdU(+) cells. **(C)** Quantification of EdU (+) cells in the subgranular zone (SGZ) of the dorsal (*p* = 0.008) and ventral (*p* = 0.0005) DG. *n* = 4 for AAV-EGFP group and n = 5 for AAV-NT-3 group. **(D)** The effect of NT-3 overexpression on cell number of doublecortin (DCX) (+) immature neurons in the ventral DG. Representative coronal images for anti-DCX immunostaining in the DG are shown. **(E)** DCX (+) cells with short or long dendrite in the SGZ (left). GCL, granule cell layer; ML, molecular layer. The numbers of DCX (+) cells with short dendrites (middle, *p* = 0.0002) and DCX (+) cells with long dendrites (right, *p* = 0.039) are shown (*n* = 5 each for AAV-EGFP group and AAV-NT-3 group). **(F)** The effect of NT-3 overexpression on cell survival in the ventral DG. Representative coronal images for anti-BrdU immunostaining in the DG are shown. The arrows represent BrdU (+) cells. **(G)** The number of BrdU (+) cells (left, *p* = 0.4138) and the ratio of NeuN (+) cells among BrdU (+) cells (right, *p* = 0.144) in the ventral DG. *n* = 5 each for AAV-EGFP group and AAV-NT-3 group. Data are expressed using dot plots and means ± standard error of mean. *p < 0.05; **p < 0.01; ***p < 0.001. n.s., not significant. Scale bars: 100 μm.

Hippocampal adult neurogenesis is a multistep process, which includes proliferation, neuronal differentiation, and survival. We evaluated the effects of high NT-3 expression on the cell proliferation. EdU was administered 2 h before sacrifice to label the proliferating cells (Figure 4A). EdU-positive cells were detected in the SGZ of the DG (Figure 4B). The number of EdU-positive cells significantly decreased in the AAV-NT-3 group in the SGZ of both dorsal and ventral DG (Figure 4C), demonstrating a suppressive effect of NT-3 on cell proliferation in the entire DG. We subsequently examined the expression of an immature neuronal marker, DCX in the ventral DG. DCX-positive cells were subclassified into those with short dendrites and those with long and branched dendrites (Figures 4D,E, left). The number of DCX-positive cells with either short or long dendrites in the SGZ significantly decreased in the AAV-NT-3 group (Figure 4E). Most of the newborn cells in the DG reportedly die during 2–3 weeks following proliferation in mice (Ueno et al., 2019). To examine the effect of high NT-3 expression on the survival of newborn cells, BrdU was administered 2–4 days before AAV injection (Figure 4A). The number of BrdU-positive cells in the ventral DG was counted at 31–33 days of cell age (Figure 4F). While no difference was observed in the survival rate between the AAV-EGFP and AAV-NT-3 groups, the ratio of NeuN-positive cells in the BrdU-positive population tended to decrease (*p* = 0.1440), although not significantly (Figure 4G). These results suggest that high NT-3 expression suppressed the early phase of the neurogenic process but had no effect on the late survival process.

### The influence of high NT-3 expression on the intracellular signaling in the DG of the hippocampus

**Figure 5:**
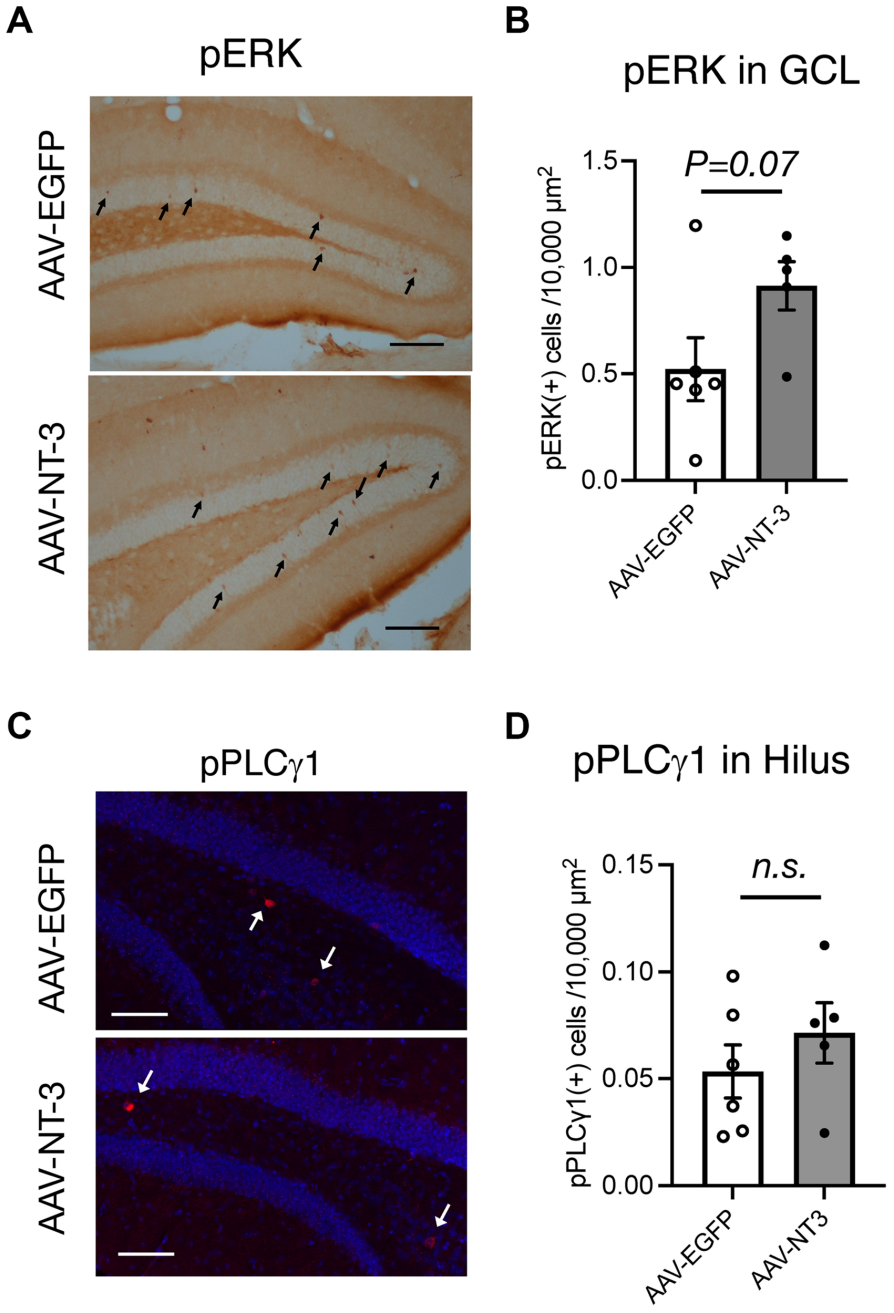
Downstream signaling of NT-3 overexpression in the ventral DG. Representative coronal images for pERK **(A)**, and pPLCγ1 **(C)** in the ventral DG are shown. Arrows in panels **(A,C)** show immune-positive cells. Scale bars: 100 μm. **(B,D)** Quantification of pERK (+) cells in the GCL (**B**, *p =* 0.0733), and pPLCγ1(+) cells in the hilus of the ventral DG (**D**, *p* = 0.3609). *n* = 6 for AAV-EGFP group and *n* = 5 for AAV-NT-3 group. Data are expressed using dot plots and means ± standard error of mean. n.s., not significant.

To explore the target cells for NT-3 signaling, we assessed the effect of NT-3 overexpression on its downstream signaling. We initially confirmed the localization of NT-3 receptor TrkC. In the Allen brain atlas database, *Trkc* mRNA expression is present in the GCL and hilus regions (Lein et al., 2007; Supplementary Figure 2A). Consistent with this, TrkC immunoreactivities were detected in the molecular layer, GCL, and hilus region in the AAV-EGFP and AAV-NT-3 group, while TrkC expression was decreased in the AAV-NT-3 group (Supplementary Figure 2B). These results indicate that NT-3 regulates downstream signaling *via* TrkC in these regions within DG, which may be subject to feedback regulation by long-term overexpression of NT-3. To determine whether NT-3 overexpression activates intracellular signaling in DG, phosphorylation of extracellular signal-regulated kinase (pERK) and phospholipase C γ1 (pPCL γ 1) were examined. pERK positive cells were scattered within the GCL. This activation tended to increase with NT-3 overexpression (Figures 5A,B). On the other hand, pPLC γ 1 positive cells were present in a small number of cells in the hilus region. This activation was not significantly altered by NT-3 overexpression (Figures 5C,D).

### Neurogenic-related gene expression changes of hippocampal DG neurons by high NT-3 expression

**Figure 6:**
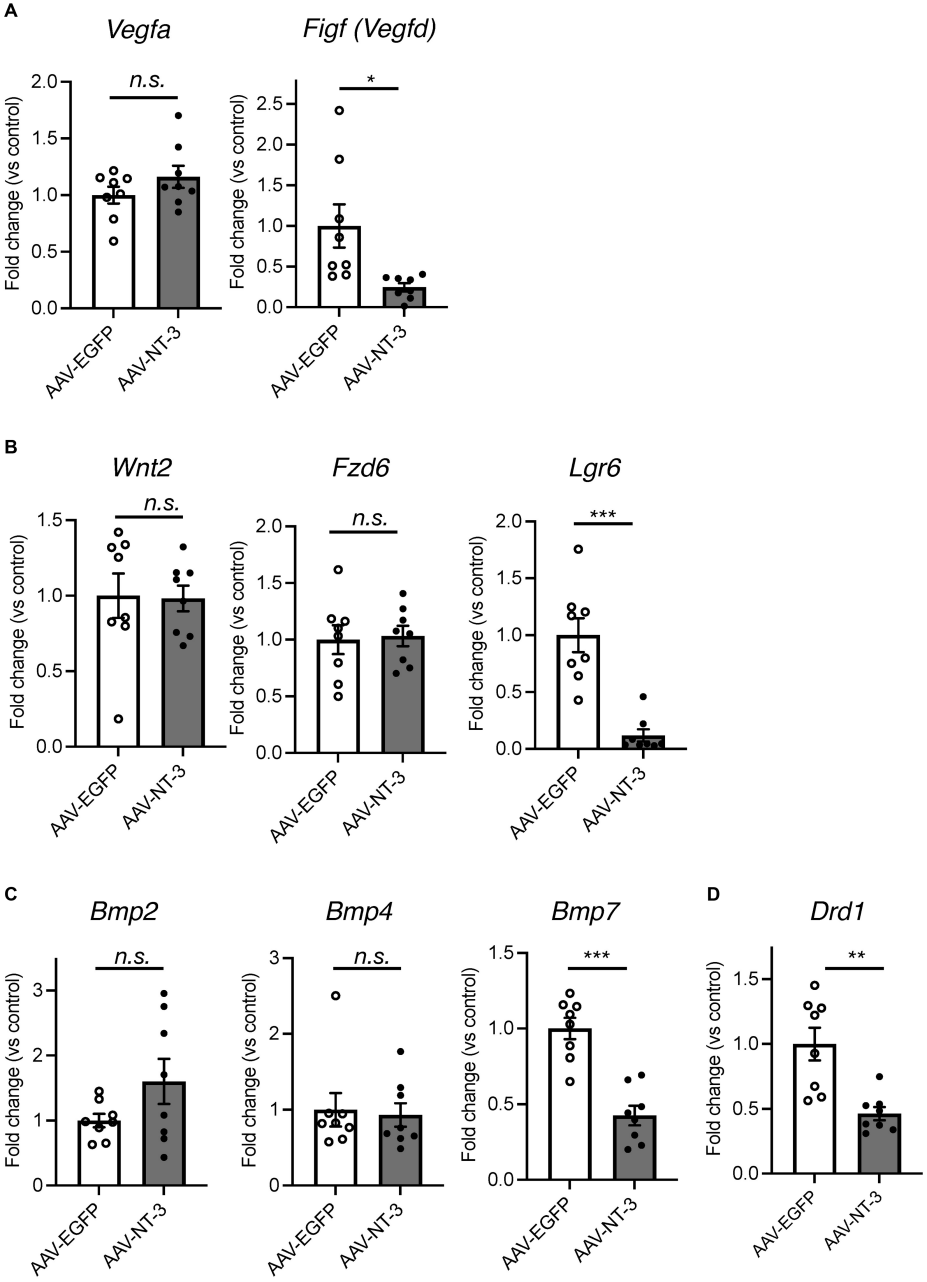
The gene expression changes of neurogenesis-related genes in the ventral DG by high NT-3 expression. **(A,B)** The effect of NT-3 overexpression on the expression of neurogenesis stimulant factors. Expression of *Vegfa* (*p* = 0.2118), *Figf* (*Vegfd*) (*p* = 0.0248), *Wnt2* (*p* = 0.9160), *Fz6* (*p* = 0.8397), *Lgr6* (*p* = 0.0003) are shown. **(C)** The expression of bone morphogenetic protein (BMP) families. Expression of *Bmp2* (*p* = 0.1325), *Bmp4* (*p* = 0.7984), and *Bmp7* (*p* < 0.0001) are shown. **(D)** The expression of D1 receptor (*Drd1*; *p* = 0.0031). *n* = 8 each for AAV-EGFP group and AAV-NT-3 group. Data are expressed using dot plots and means ± standard error of mean. *p < 0.05; **p < 0.01; ***p < 0.001. n.s., not significant.

Since our data suggested that NT-3 overexpression enhances neuronal activity and maturation in the DG, we would presume that it also regulates gene expression in an activity-dependent manner (Flavell and Greenberg, 2008). To investigate the mechanism of reduced neurogenesis by NT-3 overexpression, we examined the gene expression changes in factors related to neurogenic signals, such as vascular endothelial growth factor (VEGF) and Wnt (Lie et al., 2005; Han et al., 2015) in the DG. We found that the expression of *Vegfd (Figf)*, but not *Vegfa*, was significantly decreased by NT-3 overexpression (Figure 6A). The expression of *Wnt2* and *Fzd6*, which encode the Wnt receptor, was not altered by NT-3 overexpression, but *Lgr6*, which encodes the receptor involved in Wnt/β-catenin signaling (Liu et al., 2019), was significantly decreased in the AAV-NT-3 group (Figure 6B). We also investigated the expression of bone morphogenetic protein (BMP) families, which positively or negatively control the proliferation and neuronal differentiation in the DG during both development and adult neurogenesis (Mira et al., 2010; Mori et al., 2020). The expression of *Bmp7,* but not *Bmp2* and *Bmp4,* was significantly decreased in the AAV-NT-3 group (Figure 6C). We further found that the expression of dopamine receptor type 1 (*Drd1*), which mediates pro-neurogenic signals in the DG (Mishra et al., 2019), significantly decreased in the AAV-NT-3 group (Figure 6D). These findings may demonstrate that NT3 overexpression in the DG triggers activity-dependent gene expression that regulates the early phase of neurogenic processes.

## Discussion

In this study, we investigated the influence of high levels of NT-3 expression on the neuronal responses and neurogenic processes in the mouse hippocampal DG using the AAV expression system. We found that the expression of the mature DG neuronal markers, calbindin and IEGs, such as *Fosb* and *Fos,* was moderately increased by NT-3 overexpression. We also demonstrated that NT-3 overexpression in the DG results in the suppression of the early processes of neurogenesis, including cell proliferation. Considering the neurogenesis-related factors, the expression of *Vegfd, Lgr6, Bmp7,* and *Drd1* was significantly decreased. AAV mostly infected mature neurons in the GCL layer and cells in the hilus region of the DG. Since NT-3 is a release factor, neuronal/cell function would be affected not only in infected cells but also in surrounding ones.

During development, endogenous NT-3 is essential for the survival of peripheral sensory and sympathetic neurons (Ernfors et al., 1994; Farinas et al., 1994). In the adult central nervous system, NT-3 is highly expressed in the hippocampal DG (Smith et al., 1995). Importantly, several lines of evidence have demonstrated that NT-3 is a key regulator for the neurogenic processes, including proliferation, differentiation, and survival, in the DG of the hippocampus. A previous study demonstrated that the survival and neuronal differentiation, rather than proliferation, of the neuronal precursor cells were impaired in mice in which NT-3 expression was suppressed specifically in the brain using Nestin-Cre line (Shimazu et al., 2006). In the present study, we found that high expression of NT-3 in the DG with viral manipulation suppressed the proliferation but did not alter the survival. These findings suggest that the physiological levels of NT-3 do not affect the proliferation of neuronal precursor cells in the DG, but rather that excess amounts of NT-3 act to suppress cell proliferation. While we speculated that NT-3 overexpression can regulate cell survival, we did not observe a significant difference in the survival in NT-3 overexpression. These findings indicate that the physiological concentrations of NT-3 may be sufficient to maintain cell survival even though NT-3 is overexpressed. Interestingly, another study proposed that NT-3 can inhibit cell proliferation in the subventricular zone localized in the walls of the lateral ventricles, another neurogenic region in adults (Delgado et al., 2014). This study demonstrated that NT-3 heterozygous mice as well as endothelial NT-3 specific knockout Tie2-cre mice showed increased cell proliferation, suggesting an inhibitory effect of NT-3 on proliferation. This study may suggest that the inhibitory effect of NT-3 could occur in the SGZ of the DG in mice with NT-3 overexpression.

The role of NT-3 in neuronal differentiation remains unclear. We found a decrease in doublecortin-positive cells upon NT-3 overexpression but cannot distinguish whether this is due to inhibition of proliferation or neuronal differentiation. BrdU-labeled newborn cells at 31–33 days of cell age showed a decreasing trend in NeuN-positive neuronal differentiation, although survival remained unchanged. The effect of high expression of NT-3 on neuronal differentiation can be clarified by future analysis of surviving cells in combination with other differentiation markers for neuronal differentiation.

NT-3 overexpression resulted in an increase in Calb1 and FosB expression in the mature granule cells of the DG, suggesting that NT-3 directly or indirectly modulate their function. Hippocampal NT-3 expression is increased by chronic stress and corticosterone (Smith et al., 1995; Jiang et al., 2014). Expression of c-Fos was reportedly moderately increased in the ventral DG just after chronic social defeat stress (Anacker et al., 2018). Thus, we hypothesized that the stress-induced increase in NT-3 levels could contribute to the activation of mature neurons in the DG. A recent study reported that direct activation of the ventral DG promotes susceptibility to chronic stress (Anacker et al., 2018). Further research is warranted to clarify the influence of increased NT-3 levels in the ventral DG on emotional behaviors and stress responses.

We also found that gene expression of other types of IEGs and BDNF was enhanced by high levels of NT-3 in the DG. The cytoplasmic domains of TrkC receptors contain tyrosine phosphorylation sites that recruit various intracellular signaling molecules, including mitogen-activated protein (MAP) kinase, phosphoinositide 3-kinase (PI3K), and PLC-γ (Huang and Reichardt, 2003). Moreover, high NT-3 concentration can reportedly induce activation of adenosine 3′,5′-cyclic monophosphate (cAMP) response element binding (CREB) in cultured hippocampal neurons (Han et al., 2016), which regulate activity-dependent IEGs and BDNF expression (Esvald et al., 2022). Therefore, we investigated whether NT-3 overexpression could alter the downstream signaling pathways of NT-3-TrkC. In the present study, we detected pERK and pPLC-γ in different cells in the GCL or hilus of the DG. However, we did not observe a robust change in the downstream signals. Since our findings demonstrated that NT-3 overexpression supressed proliferation, other downstream signals or key signals could be altered by NT-3 overexpression in the SGZ of DG; however, this remains unclear. Future studies are warranted to identify the cell types in which TrkC is expressed and to identify NT-3 downstream signals according to the time course.

VEGF and Wnt signaling promote neural stem cell proliferation in the adult DG (Lie et al., 2005; Han et al., 2015). We found that the expression levels of *Vegfd* and *Lgr6* were significantly reduced by NT-3 overexpression. VEGF-D is a ligand for VEGF receptor 2 (Flk1) and VEGF receptor 3 (Flt4) (Achen et al., 1998), both of which promote neural stem cell proliferation (Segi-Nishida et al., 2008; Han et al., 2015). The expression of *Vegfd* in hippocampal neurons is regulated by nuclear calcium signaling, including Ca^2+^/calmodulin-dependent protein kinase IV (CaMKIV) (Mauceri et al., 2011). The influence of persistent NT-3 on intracellular calcium signaling needs to be investigated. LGR6 binds to R-spondins and enhances Wnt/β-catenin signaling (Gong et al., 2012), which regulates adult hippocampal neurogenesis including stem cell proliferation (Lie et al., 2005). However, the contribution of LGR6 to hippocampal neurogenesis remains largely unknown. Investigating whether LGR6 is expressed in neural stem cells, and the influence of NT-3 on Wnt/β-catenin signaling is warranted.

BMP is a factor that inhibits neural stem cell proliferation (Bond et al., 2012). Unexpectedly, the expression level of *Bmp7* in the DG was reduced by NT-3 overexpression. The proliferation of neural stem cells has been investigated in mice in which the BMP receptor Bmpr1a was ablated in the hippocampal DG of adult mice (Mira et al., 2010). The proliferation of neural stem cells was increased shortly following Bmpr1a ablation. However, 4 weeks after Bmpr1a ablation, the proliferation of neural stem cells was reduced in mice. Therefore, chronic suppression of BMP signaling may reduce neural stem cell proliferation. Chronic NT-3 expression for 4 weeks in this study may have caused a chronic decrease in the signaling of BMP7, resulting in a decrease in the proliferating cells. We also found that the expression of the dopamine receptor gene *Drd1* was reduced by NT-3 overexpression. D1 receptor agonists reportedly enhance neural stem cell proliferation by positively regulating the Wnt/β-catenin signaling pathway in the hippocampus of Parkinson’s disease rats (Mishra et al., 2019).

This study demonstrates that increased NT-3 expression in the DG possesses the potential to negatively regulate hippocampal neurogenesis *in vivo*. Since hippocampal neurogenic processes are inhibited by chronic stress (Mitra et al., 2006; Van Bokhoven et al., 2011; Campos et al., 2013) and hippocampal NT-3 expression is increased by chronic stress (Smith et al., 1995; Jiang et al., 2014), we hypothesized that NT-3 is a key neurotrophic factor that suppresses adult hippocampal neurogenesis during chronic stress. However, it should be noted that *Ntf3* expression in the overexpression group increased by more than 30-fold. The increase in the *Ntf3* expression in the DG owing to chronic unpredictable stress and 7 days of corticosterone treatment was approximately 1.5- to 2-fold (Smith et al., 1995; Jiang et al., 2014). Thus, the results of this study may not merely mimic the influence of stress-induced increase in the NT-3 levels in the DG. Thus, comparing the levels of NT-3 protein production and/or downstream signaling between stress conditions and the overexpression system used in this study is warranted. In addition, we observed that NT-3 immunostaining signals localized to cells in the hilus region. The relationship between the stress-induced increase in *Ntf3* expression and cellular accumulation at the protein level needs to be investigated. Appropriate control of NT-3 expression may reveal the role of increased NT-3 expression in stressful environments.

## Data availability statement

The original contributions presented in the study are included in the article/Supplementary material, further inquiries can be directed to the corresponding author.

## Ethics statement

The animal study was reviewed and approved by the Animal Care and Use Committee of the Tokyo University of Science (approval numbers K20009, K21007, and K22007).

## Author contributions

NK and YM designed the study, conducted the experiments, analyzed the data, and drafted the manuscript. AS conducted and analyzed the experiments. TF and SK contributed to the design and preparation of AAV. KS designed the study and drafted the manuscript. ES-N designed the study, analyzed the data, and drafted the manuscript. All authors have read and approved the final manuscript.

## Funding

This work was supported in part by MEXT KAKENHI Grants 20K07090 (ES-N), 22K20697 (KS), 21K06576 (SK), 18H05429 (TF), and 21H04812 (TF); AMED grants (JP22gm0910012 and JP22wm0425001 to TF); Kato Memorial Bioscience Foundation (to KS); Brain Science Foundation (to KS).

## Conflict of interest

The authors declare that the research was conducted in the absence of any commercial or financial relationships that could be construed as a potential conflict of interest.

## Publisher’s note

All claims expressed in this article are solely those of the authors and do not necessarily represent those of their affiliated organizations, or those of the publisher, the editors and the reviewers. Any product that may be evaluated in this article, or claim that may be made by its manufacturer, is not guaranteed or endorsed by the publisher.
