# Supplementary material for: Overexpression of NT-3 in the hippocampus suppresses the early phase of the adult neurogenic process
Source: Front Neurosci. 2023 Jul 27;17:1178555. doi: 10.3389/fnins.2023.1178555 (PMC10413268; doi:10.3389/fnins.2023.1178555)
Supplement: Supplementary file 1 [file Data_Sheet_1.pdf]

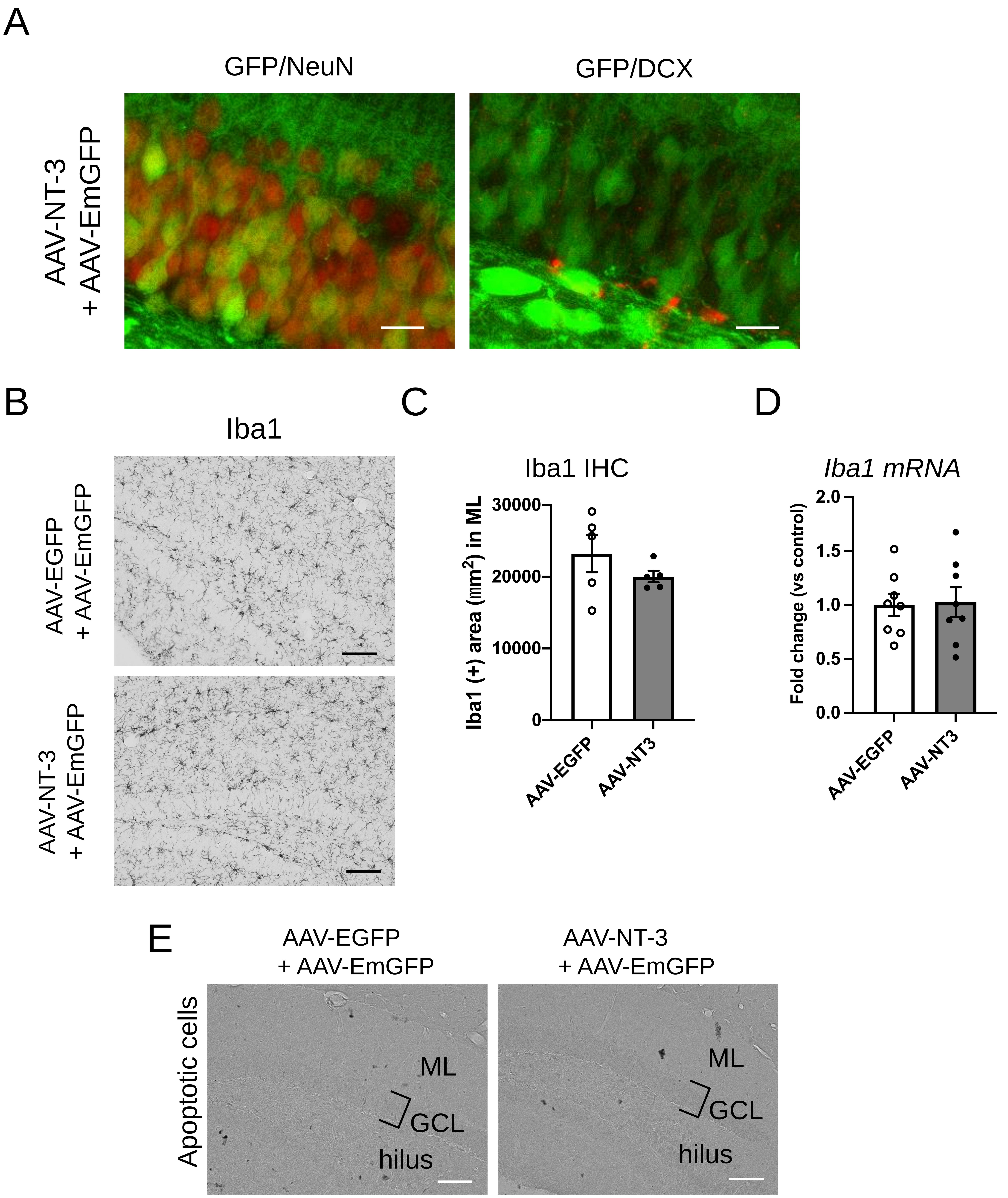

Figure S1. The infection and influence of AAV carrying NT-3 injection in the ventral DG of the hippocampus

(A) The expression of GFP (green) and NeuN (red, left) or DCX (red, right) in the GCL 4–5 weeks following AAV carrying NT-3 and EmGFP injection. Scale bars: 15 μm. (B) The expression of Iba1 in the ventral DG 4–5 weeks following AAV injection. Scale bars: 100 μm. (C) The immune-positive area of Iba1 (P=0.2939) in the molecular layer (ML). n=5 each for the AAV-EGFP group and AAV-NT-3 group. (D) The gene expression of *Iba1(Aif1)* (P=0.8868) in the ventral DG. n=8 each for the AAV-EGFP group and AAV-NT-3 group. (E) Detection of apoptotic cells in the ventral DG. Scale bars: 100 μm. ML: molecular layer; GCL: granule cell layer. Data are expressed using dot plots and means ± standard error of mean.

Figure S1

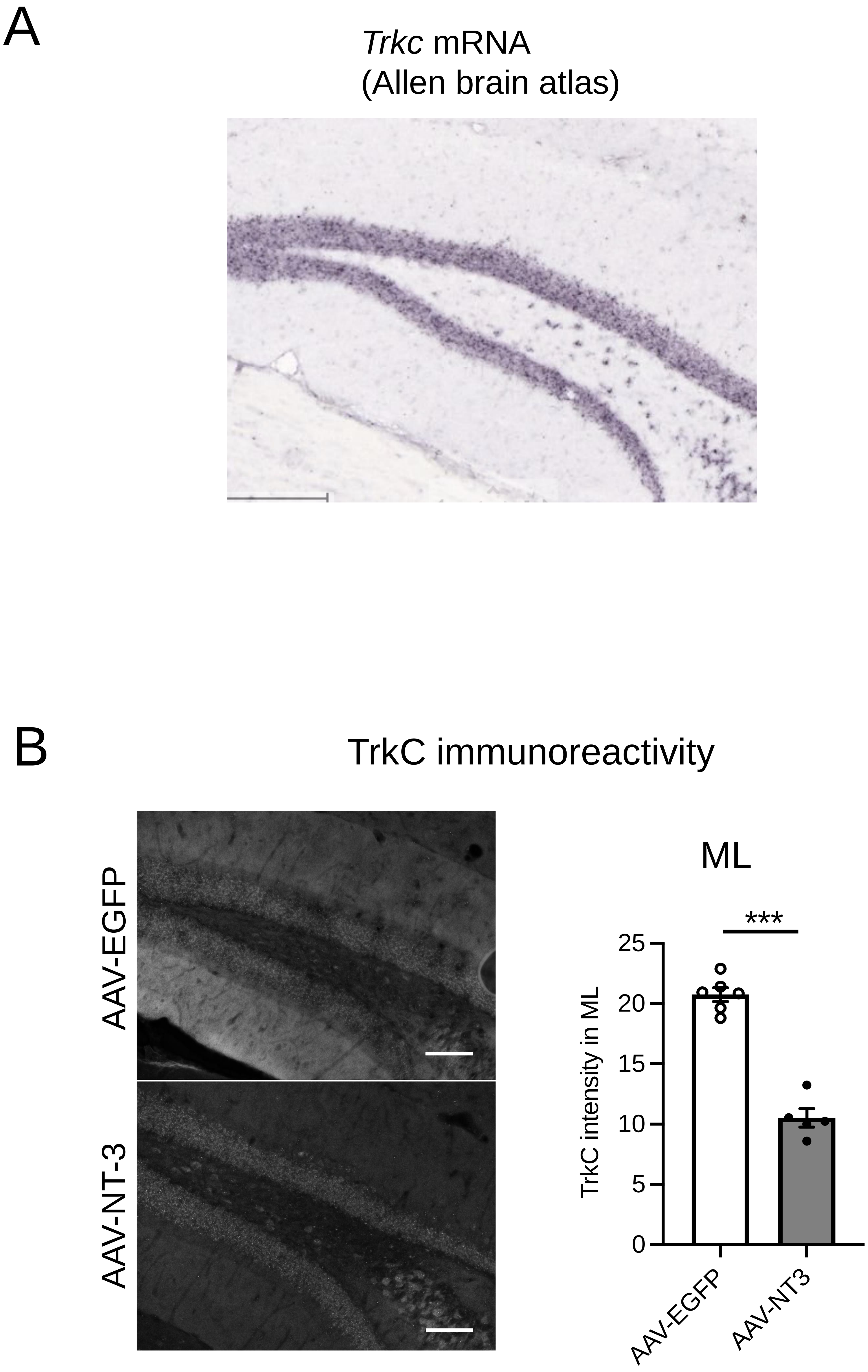

Figure S2. The expression patterns of *Trkc* mRNA in the ventral DG

(A) Expression of *Trkc* in adult mouse brain. Allen Mouse Brain Atlas, mouse.brain-map.org/experiment/show/76115738. (B) Representative coronal images for TrkC in the ventral DG are shown (left). Scale bars: 100 µm. Quantification of the intensity of TrkC immunoreactivity in the molecular layer (ML, $P<0.0001$, right). n=6 for AAV-EGFP group and n=5 AAV-NT-3 group.

Figure S2
